# Influence of the C242T Polymorphism of the p22-phox Gene (CYBA) on the Interaction between Urinary Sodium Excretion and Blood Pressure in an Urban Brazilian Population

**DOI:** 10.1371/journal.pone.0081054

**Published:** 2013-12-05

**Authors:** Roberto Schreiber, Vera Regina Bellinazzi, Andrei C. Sposito, José G. Mill, José E. Krieger, Alexandre C. Pereira, Wilson Nadruz

**Affiliations:** 1 Department of Internal Medicine, University of Campinas, Campinas, Brazil; 2 Department of Physiological Sciences, Federal University of Espírito Santo, Vitória, Brazil; 3 Laboratory of Genetics and Molecular Cardiology, Heart Institute (InCor), University of São Paulo, São Paulo, Brazil; Federal University of São Paulo (UNIFESP), Escola Paulista de Medicina, Brazil

## Abstract

**Background:**

Reactive oxygen species are implicated in the physiopathogenesis of salt-induced hypertension and the C242T polymorphism of the p22-phox gene has been associated with higher superoxide production. This study investigated the impact of this polymorphism on the relationship between urinary sodium excretion (USE) and blood pressure levels in an urban Brazilian population.

**Methods:**

We cross-sectionally evaluated 1,298 subjects from the city of Vitoria-ES, located in the Southeast region of Brazil, by clinical history, physical examination, anthropometry, analysis of laboratory parameters, USE measurement and p22-phox C242T polymorphism genotyping.

**Results:**

No significant differences in studied parameters were detected between the studied genotype groups (CC vs. CT+TT). Systolic blood pressure exhibited significant correlation with USE only in T allele carriers (r = 0.166; p<0.001), while diastolic blood pressure and hypertension status correlated with USE in both genotypes albeit more weakly in subjects with CC genotype (r = 0.098; p = 0.021 and r = 0.105; p = 0.013, respectively) than in T carriers (r = 0.236; p<0.001 and r = 0.213; p<0.001, respectively). Regression analyses adjusted for confounding factors showed that USE remained independently associated with systolic (p<0.001) and diastolic blood pressure (p<0.001) and hypertension status (p = 0.004) only in T allele carriers. Finally, higher diastolic and systolic blood pressure levels were detected in T allele carriers than in CC genotype individuals in the highest tertile of USE.

**Conclusions:**

The p22-phox 242T allele is associated with higher blood pressure levels among subjects with higher USE in an urban Brazilian population.

## Introduction

Oxidative stress, exerted by intracellular accumulation of reactive oxygen species, has been implicated in the physiopathogenesis of salt-induced hypertension [1], [2]. Nicotinamide adenine dinucleotide phosphate (NADPH) oxidase system is a major source of reactive oxygen species and is composed of multiple subunits of membrane and cytosolic components that assemble on the cellular surface to generate reactive oxygen species [3]. Among these subunits the p22-phox is highlighted as an essential membrane-associated factor that plays a crucial role in the activation and stabilization of NADPH oxidase [4].

To date, several polymorphisms of the p22-phox (CYBA) gene have been identified [5]. One of the most studied polymorphisms of this gene is the C242T, which predicts the nonconservative substitution of histidine-72 by a tyrosine residue [6] and has been shown to affect the functional activity of NADPH oxidase [7]. Although a significant heterogeneity for a modulating role of the p22-phox C242T polymorphism in the occurrence of cardiovascular disease has been reported, recent meta-analyses have suggested that the T allele is associated with increased cardiovascular risk, albeit significant associations were detected in some but not in all studied ethnic groups [8], [9]. Likewise, studies performed in urban Brazilian populations have shown that the T allele is associated with unfavorable cardiovascular phenotypes and increased NADPH oxidase activity [10], [11].

Urinary sodium excretion is usually used as the standard method for evaluation of sodium intake in clinical and epidemiological studies [12], [13]. Genetic factors have been reported to modulate the interaction between urinary sodium excretion and blood pressure [14]–[17]. In this regard, loss-of-function polymorphisms of the cytochrome P450 3A (CYP3A) and glutathione S-transferase (GST) M1 genes, which codify enzymes involved in the metabolism of reactive oxygen species, have been associated with increased blood pressure levels in subjects exhibiting high urinary sodium excretion [14], [17]. These data suggest that genetic variation in components of pathways that regulate oxidative stress may modulate the interaction between sodium intake and blood pressure levels. In accordance with this notion, Castejon et al [18] showed that the T allele of the p22-phox (C242T) polymorphism could be a genetic susceptibility factor for salt sensitivity in South-American women. Therefore, the aim of this study was to investigate the influence of the C242T p22-phox polymorphism on the relationship between urinary sodium excretion and blood pressure levels and hypertension status in an urban Brazilian population.

## Methods

### Ethics Statement

The study was conducted in accordance with the Declaration of Helsinki and the protocol was approved by the Institutional Review Board of the Espírito Santo Federal University. All participants read and signed informed consent.

### Studied Population

A cross-sectional study of risk factors for cardiovascular diseases was performed in the urban population of Vitoria, Brazil, using the WHO-MONICA project guidelines [19]. The city of Vitoria is the capital of the Espírito Santo State, which is located in the Southeast region of Brazil. A sample of 1,298 individuals was evaluated for height, weight, smoking habits, blood pressure measurements, and the use of medications. Subjects were classified as Caucasian or African descent according to a set of phenotypic characteristics (skin color, hair texture, shape of the nose, aspect of the lip, and jaw position) [13]. Exclusion criteria were age <65 years and use of any hypertensive medications.

Blood pressure was measured using a standard mercury sphygmomanometer on the left arm after 5 min rest, in the sitting position. Systolic and diastolic blood pressures were calculated from three readings with a minimal interval of 5 min. Hypertension was defined as systolic blood pressure ≥140 mmHg and/or diastolic blood pressure ≥90 mmHg. Glucose, total cholesterol, lipoprotein fractions, triglycerides, uric acid and creatinine were assayed by standard techniques in 12-h fasting blood sample. Diabetes mellitus was defined as a fasting glucose ≥126 mg/dL and/or use of hypoglycemic drugs [20]. No participant reported presence of type 1 diabetes mellitus. Body mass index was calculated as body weight divided by height squared (kg/m^2^). Height was measured in centimeters and weight in kilograms using a calibrated balance.

Urine was collected from participants during a 12-h period (from 7∶00 PM to 7∶00 AM) during the night before the clinic visit. Sodium and potassium concentrations were measured in the 12-h urine by flame photometry. All measurements were performed in the same laboratory and the same commercial kits were used in all investigations. Previous data have shown that 12-h urine collected at night can be used as a reliable tool to estimate 24-h excretion of sodium and potassium [21].

### DNA Extraction and p22-phox C242T Genotyping

Genomic DNA was extracted from leukocytes in samples of whole blood, following a standard salting-out procedure [22]. The p22-phox C242T polymorphism was analyzed by polymerase chain reaction and digestion with Rsa-I restriction enzyme as those reported previously [10]. Quality control for these assays was assessed by randomly selecting 100 samples to be re-genotyped by two independent technicians.

### Statistical Analysis

Data were analyzed using SPSS 15.0™. Descriptive statistical results are given as means ± SEM. All continuous variables presented normal distribution as assessed by the Kolmogorov–Smirnov test. A chi-square test was used to compare categorical variables and to test for Hardy–Weinberg disequilibrium. Unpaired t-test was used to compare continuous variables. Bivariate correlations between variables were examined by using Pearson correlation coefficient for normally distributed data and Spearman rank correlation coefficient for non-normally distributed data. Fisheŕs z-test was used to compare correlation coefficients. Linear and logistic regression analyses were used to evaluate the independent predictors of blood pressure levels and hypertension, respectively. Variables that exhibited significant correlation at bivariate analysis and urinary sodium excretion were included as independent variables in regression analyses. Significance was accepted if p<0.05.

## Results

Genotypic frequencies of the C242T polymorphism (42.8% for CC, 46.1% for CT, and 11.1% for TT) were in accordance with the Hardy-Weinberg equilibrium (p = 0.37; X^2^ = 0.78). The clinical, anthropometric and laboratory characteristics of studied subjects according to C242T genotypes are shown in Table 1. Given the described dominant effect of the T allele in functional and association studies [7], [10], [11], [23]–[25] TT subjects were added to CT subjects. No differences in the clinical and laboratory characteristics were detected between the genotype subgroups.

**Table 1:** Clinical features of studied subjects according to the p22-phox C242T polymorphism.

Variable	CC genotype (n = 556)	CT+TT genotypes (n = 742)
*Clinical*		
Age, years	43.5±0.4	43.3±0.4
Gender (Male/Female)	285/271	344/398
Body mass index, kg/m^2^	25.7±0.2	25.8±0.2
Systolic blood pressure, mmHg	124.0±0.8	123.9±0.7
Diastolic blood pressure, mmHg	82.2±0.6	82.2±0.5
Smoking, n (%)	135 (24)	166 (23)
Hypertension, n (%)	194 (35)	258 (35)
Diabetes mellitus, n (%)	84 (15)	128 (17)
Race (Caucasian-descent), n (%)	236 (42)	298 (41)
*Serum analysis*		
Glycemia, mg/dL	101.4±1.2	102.1±1.0
Uric acid, mg/dL	4.8±0.2	4.7±0.1
Creatinine, mg/dL	0.97±0.01	0.96±0.01
Triglycerides, mg/dL	129.0±4.0	126.6±3.5
HDL-cholesterol, mg/dL	52.3±1.6	49.8±1.1
LDL-cholesterol, mg/dL	138.9±1.7	139.8±1.4
*12 h urinary analysis*		
Sodium, mEq	95.9±2.3	95.1±1.9
Potassium, mEq	23.1±0.7	22.5±0.5

**Legend.** HDL – high-density-lipoprotein; LDL – low-density-lipoprotein.

Bivariate analysis revealed a significant correlation between systolic blood pressure and urinary sodium excretion only in allele T carriers (r = 0.166; p<0.001), while diastolic blood pressure correlated with urinary sodium excretion in both genotype groups, albeit more weakly in CC genotype than in T carriers (CC: r = 0.098; p = 0.021; T carriers: r = 0.236; p<0.001; z-score = −2.54; p = 0.011). In addition, significant correlation coefficients between hypertension status and urinary sodium excretion were detected in both genotype groups albeit also more weakly in CC genotype than in T carriers (CC: r = 0.105; p = 0.011; T carriers: r = 0.213; p<0.001; z-score = −1.98; p = 0.047). Further correlation analysis between clinical/laboratory variables and blood pressure measurements/hypertension was performed in order to determine potential confounding factors in the relationship between blood pressure/hypertension status and urinary sodium excretion in both genotype groups (Table S1).

To confirm whether the p22-phox C242T polymorphism influenced the relationship between urinary sodium excretion and blood pressure and hypertension status, linear and logistic regression analysis were performed. Noticeably, urinary sodium excretion remained independently associated with systolic and diastolic blood pressure (Table 2) and hypertension status (Table 3) only in allele T carriers after adjustment for potential confounding factors.

**Table 2:** Linear regression analysis for systolic and diastolic blood pressure.

Variable	CC genotype			CT+TT genotypes		
	*β*	*p*	R^2^	*β*	*p*	R^2^
*Dependent: SBP*			0.221			0.276
Male gender	0.132	0.003		0.117	0.004	
Age	0.238	<0.0001		0.280	<0.0001	
Race(African-descent)	–	–		0.146	<0.0001	
Body mass index	0.106	0.012		0.241	<0.0001	
Diabetes mellitus	0.200	<0.0001		0.029	0.400	
Triglycerides	0.070	0.117		0.058	0.113	
HDL-cholesterol	0.125	0.003		–	–	
LDL-cholesterol	0.060	0.129		0.006	0.860	
Creatinine	0.015	0.731		−0.002	0.953	
Uric acid	–	–		0.095	0.015	
Urinary Sodium	0.032	0.412		0.112	0.003	
Urinary Potassium	–	–		−0.075	0.044	
*Dependent: DPB*			0.284			0.264
Male Gender	0.212	<0.0001		0.164	<0.0001	
Age	0.129	0.001		0.179	<0.0001	
Race(African-descent)	–	–		0.160	<0.0001	
Body mass index	0.329	<0.0001		0.259	<0.0001	
Diabetes mellitus	0.129	0.001		−0.057	0.102	
Triglycerides	0.084	0.051		0.116	0.002	
HDL-cholesterol	0.132	0.001		−0.024	0.387	
LDL-cholesterol	–	–		−0.017	0.512	
Creatinine	0.022	0.614		−0.004	0.924	
Uric acid	–	–		0.056	0.150	
Urinary sodium	0.028	0.445		0.160	<0.0001	
Urinary potassium	–	–		−0.057	0.123	

**Legend.** SBP – systolic blood pressure; DBP – diastolic blood pressure; HDL – high-density-lipoprotein; LDL – low-density-lipoprotein.

**Table 3:** Logistic regression analysis for hypertension status.

Variable	Exp (B)	CI (95%)	p
*CC genotype (Model 1)*			
Diabetes mellitus	3.514	2.090–5.907	<0.0001
Age >43 years	2.017	1.369–2.971	<0.0001
Body mass index >25.1 kg/m^2^	1.951	1.306–2.913	0.001
Gender (Male = 0; Female = 1)	0.545	0.368–0.807	0.002
Urinary sodium >86.4 mEq/12 h	1.234	0.837–1.819	0.288
*CT+TT genotypes (Model 2)*			
Age >43 years	2.783	1.977–3.918	<0.0001
Body mass index >25.1 kg/m^2^	2.429	1.729–3.411	<0.0001
Race (African-descent)	2.058	1.447–2.925	<0.0001
Gender (Male = 0; Female = 1)	0.345	0.246–0.484	<0.0001
Urinary sodium >86.5 mEq/12 h	1.715	1.191–2.469	0.004

**Legend.** Model 1 also included triglycerides >102 mg/dL, creatinine >1.0 mg/dL and uric acid >4.6 mg/dL, while model 2 also included diabetes mellitus, triglycerides >99 mg/dL, creatinine >1.0 mg/dL, uric acid >4.6 mg/dL and urinary potassium >19.4 mEq/12 h. Only variables that exhibited significant association and urinary sodium excretion are presented in the table.

The next step was to evaluate the frequencies of the p22-phox C242T polymorphism and the clinical and laboratory features of all studied subjects according to the tertiles of urinary sodium excretion (Table 4). Increased diastolic blood pressure (86.9±0.9 vs. 83.9±0.9 mmHg; p = 0.026) and systolic blood pressure (129.1±1.3 vs. 125.5±1.2; p = 0.049) levels were detected in T allele carriers in comparison with CC genotype individuals in the highest tertile of urinary sodium excretion.

**Table 4:** Features of studied subjects according to the tertiles of urinary sodium excretion and p22-phox C242T genotypes.

Characteristics	Low (<67 mEq/12 h)		Medium (67–111 mEq/12 h)		High (>111 mEq/12 h)	
*p22-phox(C242T) Genotype*	CC	CT+TT	CC	CT+TT	CC	CT+TT
*N (%)*	184 (43)	247 (57)	178 (42)	248 (58)	194 (44)	247 (56)
*Clinical*						
Age, years	41.7±0.7	43.1±0.7	43.9±0.8	42.7±0.6	44.8±0.8	43.9±0.7
Gender (Male/Female)	75/109	93/152	97/81	97/153	113/81	154/93
Systolic blood pressure, mmHg	121.9±1.5	121.5±1.3	124.7±1.4	121.2±1.0	125.5±1.2	129.1±1.3*
Diastolic blood pressure, mmHg	80.4±1.0	79.1±0.8	82.0±0.9	80.6±0.7	83.9±0.9	86.9±0.9*
Body mass index, kg/m^2^	25.1±0.4	25.2±0.3	25.7±0.3	24.9±0.3	26.2±0.3	27.1±0.3
Smoking, n (%)	48 (26)	51 (21)	43 (24)	51 (20)	44 (23)	69 (28)
Hypertension, n (%)	53 (29)	65 (26)	61 (34)	73 (29)	80 (41)	124 (50)
Diabetes mellitus, n (%)	33 (18)	34 (14)	23 (13)	41 (16)	28 (14)	54 (22)
Race (Caucasian-descent), n (%)	84 (46)	105 (43)	67 (38)	108 (43)	85 (44)	89 (36)
*Serum analysis*						
Glycemia, mg/dL	100.6±2.2	99.5±1.2	100.9±1.8	101.9±1.7	102.4±2.1	104.8±2.0
Uric acid, mg/dL	4.5±0.1	4.7±0.1	4.7±0.1	4.4±0.1	5.2±0.4	5.0±0.1
Creatinine, mg/dL	0.97±0.16	0.93±0.01	0.96±0.01	0.94±0.01	0.98±0.01	1.00±0.01
Triglycerides, mg/dL	123.6±7.1	113.8±5.0	123.1±6.7	120.6±5.7	139.6±7.1	148.0±7.1
HDL-cholesterol, mg/dL	54.7±2.8	50.1±1.8	49.6±2.1	49.4±1.7	52.6±3.1	50.7±2.2
LDL-cholesterol, mg/dL	138.2±2.9	141.5±2.5	138.5±3.2	140.1±2.5	140.2±2.9	137.4±2.3
*12* *h urinary analysis*						
Sodium, mEq	43.4±1.1	44.6±0.9	85.7±0.9	87.7±0.8	156.7±3.2	161.5±3.3
Potassium, mEq	16.2±0.7	16.6±0.7	20.5±0.8	22.0±0.8	32.3±1.5	29.6±0.9

**Legend.** HDL – high-density-lipoprotein; LDL – low-density-lipoprotein;

* p<0.05 in comparison with CC genotype.

## Discussion

In the present report, we found that the p22-phox C242T polymorphism modulated the relationship between urinary sodium excretion and blood pressure levels in Brazilian general population. Remarkably, only in presence of the T allele, urinary sodium excretion showed an independent association with systolic blood pressure, diastolic blood pressure and hypertension status. In addition, higher diastolic and systolic blood pressure levels were detected in T allele carriers than in CC genotype individuals in the highest tertile of urinary sodium excretion. Overall, these findings indicate that the p22-phox 242T allele might be a potential marker of increased blood pressure under higher salt intake.

Although the reasons by which the 242T influenced the interaction between sodium intake and blood pressure levels were not apparent in the present study, some explanations can be proposed. High daily salt intake impairs vasodilatation and enhances vasoconstriction, resulting in reduction of regional blood flow and elevation of blood pressure in healthy and hypertensive individuals [26]. In this regard, experimental data have shown that oxidative stress in the kidney, blood vessels and hypothalamus mediates the increases in blood pressure levels induced by excess sodium intake [1], [2]. Previous data have shown that the 242T allele is associated with higher superoxide production [7]. In the present report, we did not evaluate any functional index of NADPH oxidase activity or superoxide levels in our studied population, but a previous study evaluating another Brazilian urban population showed that the 242T allele was associated with higher leukocyte NADPH-oxidase activity [11]. In addition, although other reports assessing the functional role of this polymorphism in NADPH-oxidase activity or release of reactive oxygen species have yielded conflicting results [6], cell transfection of plasmids carrying the p22-phox cDNAs with the 242T allele resulted in increased superoxide production [7]. Albeit this latter study was performed in a promyelocytic leukemia cell line, which may have very different properties than vascular or renal cell types involved in blood pressure regulation, particularly to high salt, it strengthens the notion that the 242T variant might induce gain of function for the NADPH oxidase activity. Given that p22-phox is expressed in the kidney [27] and blood vessels [28] and that higher diastolic and systolic blood pressure levels were detected in T allele carriers than in CC genotype individuals in the highest tertile of urinary sodium excretion in our sample, it could be speculated that, in the presence of the T allele, higher sodium intake would stimulate higher superoxide production, thus leading to more robust increases in blood pressure levels. In agreement with this assumption, data from other reports revealed that loss-of-function polymorphisms of the CYP3A5 and GST-M1 genes, which codify enzymes that detoxify reactive oxygen species, are also associated with higher blood pressure levels in subjects with higher urinary sodium excretion [14], [17]. However, the precise mechanisms underlying the interaction among urinary sodium excretion, p22-phox C242T polymorphism and blood pressure are unclear and further studies are required to address this issue.

Our study did not measure salt sensibility, but our main findings are in the same line of those reported by Castejon *et al* [18], who showed that the presence of T allele was associated with increased salt sensitivity in South-American women, but not in men. Differently from the aforementioned report, however, the association between the T allele and urinary sodium excretion was not influenced by gender in our sample. It is possible that such divergences were attributable to differences in sample size. For example, the study by Castejon *et al* (119 subjects-73 females/46 males) presented a remarkable smaller sample size than that of the present paper (1,298 subjects- 669 females/629 males), which strengthens the validity of our data. Despite the differences, these data indicate that the p22-phox C242T polymorphism might be a potential marker of susceptibility to blood pressure response to high sodium intake.

Some limitations to our study need to be addressed. First, we only performed a single measure of urinary sodium excretion, which might not be fully representative of sodium intake. Nevertheless, a previous study evaluating the urban population of Vitória showed that urinary sodium excretion estimated by a single 12-h urine collection had a good correlation with sodium intake assessed by questionnaires [29]. This observation suggests that although a single 12-h urine collection might be insufficient to fully characterize an individual’s sodium excretion, it might be able to reflect the average excretion of groups of subjects. Second, in order to diminish the influence of external factors on urinary sodium excretion in our analysis, we only included individuals that were not using antihypertensive medications in our protocol. Third, we cannot exclude the possibility that an allele at another locus, in strong linkage disequilibrium with the p22-phox C242T polymorphism, could account for our observed associations.

In conclusion, the present study provided novel evidence that the p22-phox 242T allele is associated with higher blood pressure levels among subjects with higher USE in an urban Brazilian population. However, further studies in other populations are warranted to confirm the current evidence.

## Supporting Information

Table S1 Bivariate correlation coefficients between blood pressure/hypertension status and clinical/laboratory variables.
(DOC)
