# Supplementary material for: Influence of the C242T Polymorphism of the p22-phox Gene (CYBA) on the Interaction between Urinary Sodium Excretion and Blood Pressure in an Urban Brazilian Population
Source: PLoS One. 2013 Dec 5;8(12):e81054. doi: 10.1371/journal.pone.0081054 (PMC3855219; doi:10.1371/journal.pone.0081054)
Supplement: Table S1 — Bivariate correlation coefficients between blood pressure/hypertension status and clinical/laboratory variables. (DOC) [file pone.0081054.s001.doc]

**Table S1.** Bivariate correlation coefficients between blood pressure/hypertension status and clinical/laboratory variables.

| Variable | CC (n=556) | | CT+TT (n=742) | |
| --- | --- | --- | --- | --- |
|  | r | p | r | P |
| *Dependent: SBP* |  |  |  |  |
| Gender (Male) | 0.166 | <0.0001 | 0.213 | <0.0001 |
| Age | 0.319 | <0.0001 | 0.317 | <0.0001 |
| Race (African-descent) | 0.056 | 0.188 | 0.136 | <0.0001 |
| Body mass index | 0.189 | <0.0001 | 0.344 | <0.0001 |
| Diabetes melllitus | 0.297 | <0.0001 | 0.212 | <0.0001 |
| Triglycerides | 0.232 | <0.0001 | 0.241 | <0.0001 |
| HDL-cholesterol | 0.138 | 0.001 | 0.029 | 0.436 |
| LDL-cholesterol | 0.117 | 0.006 | 0.132 | <0.0001 |
| Creatinine | 0.149 | <0.0001 | 0.197 | <0.0001 |
| Uric acid | 0,064 | 0.134 | 0.076 | 0.038 |
| Urinary Sodium | 0.075 | 0.079 | 0,166 | <0.0001 |
| Urinary Potassium | -0.016 | 0.714 | 0.084 | 0.023 |
| *Dependente: DPB* |  |  |  |  |
| Gender (Male) | 0.230 | <0.0001 | 0.252 | <0.0001 |
| Age | 0.215 | <0.0001 | 0.211 | <0.0001 |
| Race (African-descent) | 0.035 | 0.409 | 0.164 | <0.0001 |
| Body mass index | 0.381 | <0.0001 | 0.345 | <0.0001 |
| Diabetes melllitus | 0.256 | <0.0001 | 0.129 | <0.0001 |
| Triglycerides | 0.312 | <0.0001 | 0.267 | <0.0001 |
| HDL-cholesterol | 0.134 | 0.001 | 0.053 | 0.151 |
| LDL-cholesterol | 0.021 | 0.617 | 0.067 | 0.068 |
| Creatinine | 0.198 | <0.0001 | 0.204 | <0.0001 |
| Uric acid | 0.069 | 0.101 | 0.073 | 0.047 |
| Urinary Sodium | 0.098 | 0.021 | 0.236 | <0.0001 |
| Urinary Potassium | -0.024 | 0.567 | 0.125 | 0.001 |
| *Dependent: Hypertension* |  |  |  |  |
| Gender (Male) | 0.155 | <0.0001 | 0.250 | <0.0001 |
| Age | 0.272 | <0.0001 | 0.249 | <0.0001 |
| Race (African-descent) | 0.054 | 0.198 | 0.134 | <0.0001 |
| Body mass index | 0.256 | <0.0001 | 0.303 | <0.0001 |
| Diabetes melllitus | 0.274 | <0.0001 | 0.166 | <0.0001 |
| Triglycerides | 0.237 | <0.0001 | 0.255 | <0.0001 |
| HDL-cholesterol | -0.016 | 0.711 | 0.004 | 0.914 |
| LDL-cholesterol | 0.051 | 0.232 | 0.066 | 0.071 |
| Creatinine | 0.106 | 0.012 | 0.186 | <0.0001 |
| Uric acid | 0.281 | <0.0001 | 0.259 | <0.0001 |
| Urinary Sodium | 0.105 | 0.013 | 0.213 | <0.0001 |
| Urinary Potassium | 0.025 | 0.551 | 0.087 | 0.018 |

**Legend.** SBP – systolic blood pressure; DBP – diastolic blood pressure; HDL – high-density-lipoprotein; LDL – low-density-lipoprotein.
